# Exploring the Relationship Between Nurses’ Perceptions of Teamwork and Caring Behaviors: A Multicenter Cross‐Sectional Study in Iran

**DOI:** 10.1155/jonm/1170023

**Published:** 2026-01-31

**Authors:** Sevda Sadeghpour, Alireza Hajizadeh, Maria Namjoo Baqhini, Razie Alipour, Edris Kakemam

**Affiliations:** ^1^ Clinical Research Development Unit of Tabriz Valiasr Hospital, Tabriz University of Medical Sciences, Tabriz, Iran, tbzmed.ac.ir; ^2^ Health Information Management Research Center, Tehran University of Medical Sciences, Tehran, Iran, tums.ac.ir; ^3^ Institute for Studies in Health, Kerman University of Medical Sciences, Kerman, Iran, kmu.ac.ir; ^4^ Non-Communicable Diseases Research Center, Research Institute for Prevention of Non-Communicable Diseases, Qazvin University of Medical Sciences, Qazvin, Iran, qums.ac.ir

**Keywords:** caring behaviors, hospital, nursing, teamwork

## Abstract

**Background:**

Despite the important role of teamwork in ensuring high‐quality nursing care, little research has been conducted on nursing teamwork and its relationship with nurses’ caring behaviors, especially in developing countries.

**Aim:**

This study aimed to determine the relationship between the perceptions of teamwork and caring behaviors among nurses working in hospitals in northwest Iran.

**Methods:**

This cross‐sectional study was conducted among 401 nurses who were recruited through a simple random stratified sampling method in four public and private hospitals in Tabriz, northwest Iran. Data were collected using questionnaires that assessed demographic characteristics, the Teamwork Attitudes Questionnaire, and the Caring Behavior Inventory (CBI‐24). Data were analyzed through descriptive statistics, Pearson correlation coefficient, and univariate and multivariate linear regression analyses using SPSS‐26 software.

**Results:**

The mean values obtained in the study indicate that the participants had acceptable levels of teamwork (3.64 ± 0.62) and caring behaviors (5.10 ± 0.74). A positive relationship was observed between the teamwork and the caring behaviors score of the participants (*r* = 0.555; *p* < 0.01), and after controlling for confounder variables, team structure (*β* = 0.129), mutual support (*β* = 0.250), and communication (*β* = 0.304) explained 35.6% of the variance in caring behaviors (*F* = 25.56; *p* < 0.01; adjusted *R* ^2^ = 0.356).

**Conclusion:**

The results demonstrate that enhancing nursing teamwork, particularly in terms of team structure, mutual support, and communication, can be an effective strategy for promoting caring behaviors in hospitals. Strengthening nursing teamwork should be emphasized as one of the main priorities by all stakeholders, including clinical nurse leaders, managers, and instructors.

**Implication for Nursing Management:**

The present study has implications for nurses, nursing and hospital administrators, nursing schools, and the health system at the macro level. Through inclusion in accreditation programs, reviewing educational curricula can help strengthen teamwork among nurses. By addressing these implications, stakeholders at all levels can benefit from the powerful link between teamwork and compassionate caring behavior of nurses in the hospitals.

## 1. Introduction

Teamwork, in this context, refers to cultivating in team members a cohesive set of knowledge, skills, and attitudes essential for fulfilling their designated roles within the team [1]. The core elements of this concept encompass leadership, situational awareness, supportive behaviors, and clear communication [1–3]. Research suggests that dysfunctional teams significantly contribute to diminished patient safety. Consequently, fostering a strong, global healthcare team is paramount to improving patient safety [4–6]. Also, effective teamwork among multidisciplinary members directly translates to enhanced patient safety [7, 8]. Teamwork among nurses in a clinical setting is generally beneficial, but it also has potential downsides. These downsides can include impaired communication, increased likelihood of error due to misunderstandings or unclear roles, and interprofessional staff conflict [9].

Given the increasing complexity of clinical settings, the need for strong teamwork has been emphasized as a primary necessity for improving the quality of care. In this regard, Greco emphasizes the inherently multidisciplinary nature of healthcare delivery, highlighting the crucial role of physician–nurse–paramedic collaboration in ensuring patient safety [10]. This collaborative effort forms the bedrock of safe and effective care. Additionally, research suggests that robust nursing teamwork is associated with a significant reduction in adverse events and medical errors between nurses, alongside a rise in reported incidents to patient safety units [11–13]. The positive impact of teamwork extends to minimizing missed care, as evidenced by various studies [14, 15]. Furthermore, a Saudi Arabian study involving nurses found that collaborative teamwork enhances the quality of medical care, strengthens patient safety, and diminishes the occurrence of side effects [8].

Considering nursing teamwork with caring behaviors in the hospital is also important in providing safe and quality care to patients. In this regard, teamwork holds the potential to significantly influence the quality of nursing care behaviors [16]. It is important to acknowledge that dysfunctional teams can also lead to a decline in care quality [17, 18]. Nursing care forms a substantial component of overall patient healthcare delivery [16]. By prioritizing patient perceptions over rote care behaviors, nurses become better equipped to provide the necessary support and tailor comprehensive nursing care that addresses individual patient needs [19, 20]. Research has shown that such caring behaviors significantly enhance a patient’s sense of security, reduce anxiety, and foster a strong bond between healthcare providers and patients. These caring behaviors encompass the actions and approaches nurses employ to deliver physical and emotional care. Through these actions, patients experience a strengthened sense of safety and security, which can even contribute to a faster recovery trajectory [21, 22]. A core consideration in care delivery is the consistent pursuit of high‐quality care that meets patient satisfaction [21–23]. Accordingly, addressing a patient’s individual needs forms the cornerstone of effective nursing care [24, 25]. Ultimately, the nurse’s primary objective is to provide high‐quality patient care. In essence, quality care is both the right of all patients and the responsibility of every nurse committed to a caring approach [21, 26].

Nurses, as the backbone of medical care teams, hold a crucial position due to their direct and continuous interaction with patients. In this context, understanding the influence of teamwork on care behaviors becomes paramount. While prior research in other countries has explored this association to a limited extent [16], no such investigation has been undertaken amongst Iranian nurses. Therefore, the present study aims to bridge this gap in knowledge by examining the relationship between the perceptions of teamwork and caring behaviors among nurses working in hospitals within Tabriz, Iran. Based on this premise, we hypothesized that positive teamwork would be positively associated with better caring behaviors. The findings hold significant value for hospital and nursing administrators, informing the development of interventions to strengthen teamwork amongst nurses. Ultimately, these efforts can lead to improved quality of care behaviors, promoting service excellence and patient safety.

## 2. Materials and Methods

### 2.1. Study Design

This study employed a multicenter cross‐sectional research design. Data collection occurred between June and August of 2024. In this study, Strengthening the Reporting of Observational studies in Epidemiology (STROBE) for cross‐sectional studies was employed in describing the design and results.

### 2.2. Setting

The study setting encompassed four hospitals, both public and private, located in Tabriz, Iran. Tabriz is a city in the northwest of Iran and is one of the most populous cities in the country. There are more than 20 public and private hospitals in Tabriz that provide a variety of health services to the residents of this city and the surrounding cities. We selected four hospitals randomly from these hospitals.

### 2.3. Study Population

The target population included 2270 nurses working in the selected hospitals. The number of nurses working in public hospitals was almost double that of private hospitals. The criteria for entering the study include nurses who had at least 6 months of work experience and were working full‐time because new nurses need to adapt to both work conditions and human relationships. Generally, it takes between six and 12 months for a new nurse to become oriented and adapt to a new workplace. Nurses who worked in two hospitals at the same time and worked in paraclinical and administrative departments were excluded from the study. Furthermore, nurses on maternity leave and annual leave, and nurses with a serious illness during the data collection period, were excluded from the study.

### 2.4. Sample Size and Sampling Method

The sample size was calculated using G∗power 3.1.9.4 according to previous research (correlation coefficient = 0.295) [16]. The minimum sample size required for multiple regression analysis was 331 based on a power of 95%, a significance level (*α*) of 0.01, *ρ* ^2^ of 0.087 (effect size = 0.095), and 9 tested predictor variables (five teamwork subscales and four participants’ characteristics). Based on the above, 413 copies of the questionnaire were distributed, considering the dropout rate of 25%, and 401 were retrieved and used in the final analysis.

A simple random stratified sampling method was used to recruit the nurses. The total number of nurses working in each hospital was taken from the human resource management office of the hospital and then we divided the sample size proportionally between the four hospitals. Finally, a computer‐generated simple random sampling technique was used to select participants.

### 2.5. Study Instruments

A self‐report questionnaire with the sample’s sociodemographic and work‐related characteristics, teamwork, and caring behavior questionnaires was used for data collection.

1. Sociodemographic and work‐related characteristics: this section included age, gender, marital status, work experience in nursing, education level, workplace department, position, workload (weekly working hours), participation in teamwork courses, and hospital ownership.
2. Team‐STEPPS Teamwork Perception Questionnaire (T‐TPQ): this instrument, developed by the American Agency for Health Care Research and Quality in 2006 [27, 28], is a well‐established tool for measuring teamwork within healthcare settings. The T‐TPQ comprises 35 items categorized into five dimensions of teamwork, including team structure (7 items), team leadership (7 items), situation monitoring (7 items), mutual support (7 items), and communication (7 items).
 Participants were asked to express their level of agreement with each statement on a 5‐point Likert scale ranging from 1 (*completely disagree*) to 5 (*completely agree*). A high score on the scale indicates that the attitudes of nurses toward teamwork characteristics are positive. A mean score of more than three indicates acceptable teamwork. To ensure the instrument’s cultural relevance, the questionnaire was translated into Farsi by the researchers [29]. The validity and reliability of T‐TPQ were confirmed among Iranian nurses with a Cronbach’s alpha coefficient of 0.88 [29]. In this study, the Cronbach’s alpha coefficient of the total T‐TPQ was 0.95 and those for structure, team leadership, situation monitoring, mutual support, and communication were 0.83, 0.93, 0.88, 0.84, and 0.86, respectively.
3. Caring Behavior Inventory (CBI‐24): The study employed the CBI‐24 instrument, a revised version developed by Wu et al. [30], to measure caring behaviors exhibited by nurses. This 24‐item tool is a refinement of a larger 75‐item questionnaire originally designed by Wolf et al. [31]. The CBI‐24 assesses caring behaviors across four key dimensions:
  • **Respectful deference to others (6 items):** this subscale captures the nurse’s respectful and courteous interactions with the patient during care delivery.
  • **Assurance of human presence (8 items):** these items evaluate the nurse’s ability to instill a sense of encouragement, confidence, and calmness in the patient, thereby reducing anxiety.
  • **Positive connectedness (5 items):** this subscale focuses on the nurse’s communication style, aiming to assess the creation of a sense of solidarity, empathy, and connection with the patient.
  • **Professional knowledge and skills (5 items):** this dimension measures the nurse’s specialized knowledge and skills relevant to performing treatments and providing competent patient care.

Participants responded to each statement on a 6‐point Likert scale ranging from 1 (*never*) to 6 (*always*). Higher scores indicate a greater degree of demonstrated caring behaviors. The Farsi translation of the CBI‐24, validated by Rafiei et al. in 2008 [32], was used in this study. Their investigation confirmed both the instrument’s validity and reliability, with a Cronbach’s alpha coefficient of 0.92 [32]. In this study, the Cronbach’s alpha value was found to be 0.91.

### 2.6. Data Collection

To facilitate data collection, an introductory letter was issued by the University’s Research Vice Chancellor, granting access to the selected hospitals. Two trained data collectors visited the hospitals in person to administer the questionnaires. After securing coordination with nursing managers and department supervisors, the data collectors distributed the questionnaires among the nurses. Before participation, enumerators received comprehensive explanations regarding the study objectives and the process of completing the questionnaires. Written informed consent was obtained from each nurse, ensuring their autonomy to participate and the confidentiality of their responses. Following questionnaire distribution, nurses were granted 5–7 days for completion. During this period, data collectors revisited the wards to collect completed questionnaires and provide reminders to outstanding participants. The questionnaires were completed in 5–10 min.

### 2.7. Ethical Consideration

Before commencing the study, ethical approval was formally obtained from the Ethics Committee of Tabriz University of Medical Sciences (IR.TBZMED.REC.1403.429). Before delivering questionnaires to the nurses, the aim of the study was explained to them. Nurses were told that they could ask any questions about how to complete the questionnaire. Written informed consent in simplified Persian was obtained from all participants, explicitly stating voluntary participation, the right to withdraw, and data confidentiality. All data collected was securely stored. Paper forms, including consent forms, were kept in a secure location. All methods in this study were implemented in accordance with the institutional research committee’s ethical standards, as well as the Helsinki Statement (2013).

### 2.8. Data Analysis

Following questionnaire collection, all data were analyzed using SPSS‐26 software. Descriptive statistics were employed to summarize key variables, including teamwork, caring behaviors, and demographic characteristics. These descriptive results were presented in the form of mean, standard deviation (SD), frequency, and percentage. To examine the research hypotheses, inferential statistical methods were utilized. Before conducting these analyses, data normality was confirmed for all variables by the skewness, kurtosis scores, and the Kolmogorov–Smirnov test [33]. Pearson’s correlation coefficient was employed to evaluate the relationships between teamwork and caring behaviors. First, we conducted a univariate regression analysis identifying variables with *p* values < 0.2. Then, the multivariate linear regression analysis was used to investigate whether the dimensions of nursing teamwork could predict caring behaviors (all variables were tested by the univariate model at the significance level of 0.2 were entered into the multivariate model). Assumptions of the multivariate model, including autocorrelation of the model residuals and homoscedasticity were tested with the Durbin–Watson and Breusch–Pagan tests. Furthermore, multicollinearity was tested using tolerance and variable inflation factors. For the multivariate model, the Durbin–Watson was 1.73, which confirms the absence of positive or negative autocorrelation of the model residuals. The range of the tolerance values was between 0.351 and 0.949. As a result, the multicollinearity assumption was not violated (tolerance values > 0.2). Furthermore, none of the variance inflation factor values was greater than 5, indicating that there was no multicollinearity (1.05–2.85). Finally, the Breusch–Pagan test did not reject homoscedasticity of the final model (*p* = 0.47). The effects of the independent variables were expressed in terms of unstandardized B and standardized regression coefficients (*β*). The amount of variance explained in the model was reported in terms of *R* ^2^. A significance level of 0.05 was adopted for all statistical tests.

## 3. Results

### 3.1. Participants’ Sociodemographic and Work‐Related Characteristics

Table 1 shows the characteristics of the participants in this study. Of the 413 nurses, 401 returned completed surveys with a response rate of 97.1%. The sample consisted of 401 nurses with a mean age of 30.9 years (SD = 8.1 years). Most participants were women (66.1%) and 54.6% of them were single. Most of the participants (85.0%) were in a nurse position and had a 4‐year bachelor’s degree (78.6%). Over 60% of the nurses worked in governmental hospitals, while 37.9% worked in general wards (e.g., medical/surgical units). The majority of nurses had a heavy workload (76.3%). A total of 62.3% of the nurses stated that they had previously taken part in teamwork courses.

**Table 1:** Characteristics of participants (*n* = 401).

Demographic characteristics	Frequency	%^†^
Gender		
Male	136	33.9
Female	265	66.1
Marital status		
Single	219	54.6
Married	182	45.4
Age (years)		
< 31	239	59.6
31–39	111	27.7
≥ 40	51	12.7
Education level		
Bachelor degree	315	78.6
Master or PhD degree	86	21.4
Years of experience in nursing		
≤ 5 years	215	53.6
6–10 years	101	25.2
> 10 years	85	21.2
Position		
Nurse	34	85.0
Nursing manager	60	15.0
Workplace department		
ICUs	140	34.9
Emergency	109	27.2
General wards	152	37.9
Participation in teamwork courses		
Yes	250	62.3
No	151	37.7
Workload		
≤ 44 h/week	131	32.7
> 44 h/week	270	76.3
Hospital ownership		
Governmental	256	63.8
Private	145	36.2

^†^Percentage.

### 3.2. Teamwork and Caring Behaviors of the Nurses

The mean values of overall teamwork, overall CBI, and their subscales are provided in Table 2. The mean of the total caring behaviors score of the nurses was 5.11 (SD = 0.75), and the highest mean values in caring behavior subscales were found in “respectful deference to others” (mean = 5.15 and SD = 0.79) and in “assurance of human presence” (mean = 5.15 and SD = 0.78). The mean score on the total teamwork was 3.64 (SD = 0.62). At the subscale level, the highest mean values were found in “team structure” (mean = 3.88 and SD = 0.70).

**Table 2:** Levels of teamwork and caring behaviors.

Variables	Range score	Mean	SD^†^
Total teamwork	1–5	3.64	0.62
Team structure	1–5	3.88	0.70
Team leadership	1–5	3.63	0.95
Situation monitoring	1–5	3.58	0.78
Mutual support	1–5	3.78	0.66
Communication	1–5	3.33	0.56
Total caring behaviors	1–6	5.10	0.74
Respectful deference to others	1–6	5.15	0.79
Assurance of human presence	1–6	5.15	0.78
Positive connectedness	1–6	5.00	0.83
Professional knowledge and skills	1–6	5.11	0.75

^†^Standard deviation.

### 3.3. Correlation Among Teamwork Subscales and Caring Behaviors

The results of this study revealed that total teamwork had a significant and positive relationship with overall caring behaviors (*r* = 0.555; *p* < 0.001). Also, it was found that there were statistically positive correlations between team structure, team leadership, situation monitoring, mutual support, communication scores, and total caring behaviors scores of nurses (*r* = 0.421–0.546; *p* < 0.001). See Table 3 for further details.

**Table 3:** Correlation of teamwork and caring behaviors scores.

Variables	Total teamwork	Team structure	Team leadership	Situation monitoring	Mutual support	Communication
Overall caring behaviors	0.555^∗∗^	0.471^∗∗^	0.426^∗∗^	0.421^∗∗^	0.517^∗∗^	0.546^∗∗^
Respectful deference to others	0.545^∗∗^	0.461^∗∗^	0.413^∗∗^	0.411^∗∗^	0.507^∗∗^	0.549^∗∗^
Assurance of human presence	0.474^∗∗^	0.392^∗∗^	0.359^∗∗^	0.339^∗∗^	0.470^∗∗^	0.480^∗∗^
Positive connectedness	0.544^∗∗^	0.468^∗∗^	0.424^∗∗^	0.432^∗∗^	0.478^∗∗^	0.521^∗∗^
Professional knowledge and skills	0.535^∗∗^	0.456^∗∗^	0.412^∗∗^	0.407^∗∗^	0.502^∗∗^	0.515^∗∗^

^∗∗^ *p* < 0.001.

### 3.4. The Effect of Teamwork Dimensions on Caring Behaviors

The results of univariate and multivariate regression models are presented in Table 4. In the univariate model, sex, education level, hospital ownership, and participation in teamwork courses had *p* <  0.20. The results showed that *F* = 25.56 and *p* <  0.001, indicating that the multiple linear regression equation was statistically significant. Based on the model, teamwork dimensions were able to predict 35.6% of the changes in the total score of caring behaviors (adjusted *R* ^2^ = 0.356) and there was a positive relationship between overall caring behaviors and teamwork dimensions. Higher scores on team structure were significant predictors of higher care behaviors among nurses (*β* = 0.129; *p* < 0.038). Also, one unit increase in the score of mutual support led to a 25% increase in the caring behaviors rate (*β* = 0.250; *p* < 0.001). Finally, a unit increase in the score of communication was associated with an increase of 30.4% in caring behaviors (*β* = 0.304; *p* < 0.001).

**Table 4:** Univariate and multivariate regression models of factors associated with overall caring behaviors.

Variables	Univariate model			Multivariate model		
	*B*	*β*	*p*	*B*	*β*	*p*
*Sociodemographic and work-related characteristics*						
Age	0.002	0.024	0.625			
Years of experience in nursing	0.006	0.050	0.317			
Marital status (ref: single)	−0.014	−0.009	0.851			
Position (ref: nurse)	0.036	0.017	0.732			
Workload (ref: ≤ 44 h/week)	0.013	0.008	0.869			
Workplace department (ref: ICUs)						
Emergency department	−0.079	−0.011	0.405			
General wards	−0.016	−0.011	0.852			
Sex (ref: male)	0.173	0.110	0.028^∗^	0.169	0.108	0.009^∗∗^
Education level (ref: Bachelor degree)	−0.212	−0.117	0.019^∗^	−0.071	−0.039	0.346^∗∗^
Hospital ownership (ref: governmental)	0.212	0.137	0.006^∗∗^	0.026	0.016	0.706
Participation in teamwork courses (ref: yes)	−0.194	−0.126	0.011^∗^	0.083	−0.054	0.199

*Subscales of teamwork*						
Team structure	0.504	0.471	< 0.001^∗∗^	0.138	0.129	0.038^∗^
Team leadership	0.332	0.426	< 0.001^∗∗^	0.074	0.095	0.120
Situation monitoring	0.400	0.421	< 0.001^∗∗^	0.116	0.122	0.072
Mutual support	0.579	0.517	< 0.001^∗∗^	0.279	0.250	< 0.001^∗∗^
Communication	0.724	0.546	< 0.001^∗∗^	0.403	0.304	< 0.001^∗∗^
*F* (*p*)				25.56 (< 0.001)		
Adjusted *R* ^2^				0.356		

^∗^ *p* < 0.05.

^∗∗^ *p* < 0.01.

## 4. Discussion

Today, effective teamwork is considered a crucial tool for ensuring patient‐centered care and safe, high‐quality patient care. This study was designed to investigate the relationship between the perceptions of teamwork and caring behaviors among nurses working in public and private hospitals in Tabriz, Iran. The results of this study indicate that there is a significant relationship between nursing teamwork and caring behaviors in Iranian hospitals. Among the dimensions of teamwork, team structure, mutual support, and communication explained 35.6% of the variance of caring behaviors.

Based on the results, the mean score of teamwork and caring behaviors among nurses was equal to 3.64 out of 5 and 5.10 out of 6, respectively. The overall mean score of nursing teamwork in our study is lower than that in other similar studies conducted in other countries [14, 34–36]. With a similar questionnaire, the overall percentage of caring behaviors of nurses in research in Ethiopia (2023) was 53.3% [37]. Also, the average score of the total caring behaviors among nurses working in surgical clinics in Turkey (2019) was 4.95 ± 0.54 [16]. The variation in the results of the studies may be due to the scope of the study area and other reasons. Most of the similar studies have been conducted in single and public hospitals, but our study was conducted in public and private hospitals. Also, the diversity of results may be due to differences in assessment tools and analysis methods. There are differences between the standards of care in private and public hospitals in Iran that may harm nurses’ teamwork and caring behaviors. Public hospitals face mostly challenges, such as patients’ overcrowding, shortage of nursing staff, newly graduated nurses, lower nurse‐to‐patient ratios, weak communication between nurses and physicians, and low responsiveness and quality of nursing services, compared with private hospitals [38, 39]. However, Khoshakhlagh’s study demonstrated that the patient safety culture in public hospitals was higher than that in private ones [40].

The results of the study showed that there was a statistically positive correlation between team structure and caring behaviors. Similar to our study, it was found that there is a significant positive correlation between the subscale of nurses’ team structure and the subgroups of professional behavior in the research of Çelik et al. [16]. It means that clear goals, roles, and responsibilities, enough skills and information, and accountability can lead to cohesion and unity, which in turn play an essential role in the behavioral care of nurses in the hospital. Membership in well‐structured teams, which show clarity in team and individual goals, meet regularly, and recognize diverse skills of their members, is known to improve patient safety and quality of care [41, 42]. Working in a tight team can lead to reduced burnout [43]. In addition, a study carried out in England revealed that hospital employees who work in well‐structured teams have the highest levels of job satisfaction and the least intention to leave and job stress [44], which in turn, these factors affect caring behaviors [45–47]. In the hospital, nurses are strongly advised to have structured cooperation to reach effective teamwork [13]. On the other hand, team cohesion is a critical factor in the provision of high‐quality care [48].

Our results showed that nurses’ perception of positive mutual support is linked with better nurses’ caring behaviors. This result aligns with the main assumption of the Nursing Work–Life Model (NWLM) domains as important in supporting nurses’ work environment to enhance the quality of nurses’ caring behaviors [49]. The NWLM model proposed by Leiter and Laschinger explains how nursing management can create work environments that support professional nursing practice and ensure high‐quality patient care [49]. Furthermore, the results are consistent with a study conducted in Malaysia among nurses, which suggested that nursing managers’ ability, leadership, and support of nurses have a positive effect on nurses’ caring behaviors [50]. Mutual support may be important in providing care because low mutual support indicates poor interpersonal relationships and a lack of communication among nurses [51]. Mutual support, which is an important concept of teamwork, can be a solution to improve patients’ safety during nurses’ work overload. It means helping other team members to carry out their tasks and asking them to help whenever necessary, providing timely and constructive feedback, effectively advocating for the patient when something threatens his/her safety, and using appropriate techniques to resolve interpersonal conflicts are other aspects of mutual support [52]. Evidence indicates that the importance of mutual support in the Iranian healthcare system is underestimated, as the attitudes toward mutual support are poor [53], and “seeking help from others” is considered a negative characteristic of team members [52]. It may be argued that the lack of attention to seeking help from others can be rooted in Iranian cultural issues that make people less likely to do it. A culture of mutual support is both an individual and an organizational issue. Organizations with good teamwork have been reported to have positive clinical outcomes. Strong multiprofessional collaboration improves patient outcomes [54]. Furthermore, organizations with better mutual support reported lower staff retirement intentions [55]. Previous studies indicate that higher mutual support is associated with high patient safety and a lower probability of burnout and staff turnover [51, 56]. In addition, Kakemam et al. found that nurses with higher mutual support levels were more likely to report adverse events [13].

The results of this study revealed that communication was a positive predictor of care behaviors. These results can be interpreted as establishing that sufficient communication positively affects care behavior. The results are similar to previous studies of nurses in Turkey [16, 57]. Communication is the cornerstone of nursing and has a critical role in nursing practice and caring behaviors in hospitals. Communication with patients and families is an essential component of high‐quality care in serious illness [57]. Lack of establishing professional communication leads to failure in care [58]. Furthermore, Avallin et al. suggested that communication can be a vital tool to avoid missed nursing care and address the critical need for nursing managers to restore the fundamentals of care [59]. Active communication may reduce conflict, lessen stress among team members, and promote healthy communication [60, 61]. In line with the importance of communication in caring behaviors, the results of Rivera’s research showed that if the needs of patients are correctly defined, the relationship between nurse and patient will strengthen, and as a result, behavioral care and quality of care will improve [62]. Examples of the growing clinical evidence of improved patient outcomes and reduced harm resulting from effective teamwork, especially through its communication dimension, are reported [63]. Jones et al. stated in their study that there is insufficient communication between the nurse and patient due to an insufficient number of nurses and too many patients [64].

### 4.1. Limitations

This study has several limitations that should be considered when interpreting the findings.

First, we applied a cross‐sectional design, which makes it difficult to a causal inference. This design provides a snapshot of the extent to which the variables of the study are related without introducing the complexities of temporal flows that might distort relationships. Therefore, future research should use robust designs, including longitudinal and interventional designs. Second, the study relied on self‐report data, which could be skewed by response biases, including social desirability bias or recall bias; observational methods will need to be added in future studies. Third, the sample for the current study comes from private and public hospitals. The differences in standards of care, work environment conditions, and cultural issues, including organizational, teamwork, and patient safety culture in the hospitals under study, may affect the findings. The future research may explore cultural and structural influences on teamwork and care behaviors. Finally, the research population of this study was limited to nurses, and since providing care is teamwork in the hospital, it is necessary to overcome this research limitation with a large sample size and with the participation of other health service providers (e.g., physicians and paramedical staff).

### 4.2. Implication for Nursing Management

The results of this study show a positive and significant relationship between nurses’ perceptions of teamwork and their caring behaviors in Iranian hospitals. Therefore, the present study has theoretical, practical, and broader implications for various stakeholders, especially in the field of nursing in Iran. In this regard, nurses working in hospitals must consciously move beyond seeing teamwork as a passive requirement and actively participate in collaborative practices. Nurses need to strengthen teamwork to have positive outcomes in their care behaviors to provide quality health services to patients. For nursing and hospital administrators, the results of this study provide a strong, evidence‐based rationale for investing in and strengthening team dynamics as a constructive strategy for improving patient‐centered care in the hospital. Generally, the foundation for effective teamwork is laid during nurses’ professional education. Therefore, nursing curricula should evolve to prepare future nurses for a collaborative work environment. At the macro level (health system), the results argue in favor of systemic changes to include effective teamwork as a standard in hospital accreditations to help strengthen nurses’ caring behaviors.

## 5. Conclusion

The results of the current study demonstrate that enhancing nursing teamwork, particularly in terms of team structure, mutual support, and communication, can be an effective strategy for promoting caring behaviors in hospital settings. These results have implications for all healthcare stakeholders and provide a basis for specific interventions and educational initiatives designed to enhance teamwork with the ultimate goal of improving caring behaviors among nurses. In this regard, we recommended that clinical nurse leaders, administrators, and instructors focus on strengthening nursing teamwork, especially the dimensions of mutual support, communication, and team structure to improve caring behaviors. They can actively promote nursing teamwork through training and education, organizational (re)design, using structuring, triggering, and facilitating tools.

## Conflicts of Interest

The authors declare no conflicts of interest.

## Author Contributions

Edris Kakemam and Sevda Sadeghpour conceptualized the initial idea and developed the protocol. Edris Kakemam, Sevda Sadeghpour, and Alireza Hajizadeh collected the data. Maria Namjoo Baqhini and Razie Alipour performed the formal analysis with contributions from Edris Kakemam. Edris Kakemam, Razie Alipour, and Sevda Sadeghpour wrote the original draft. Razie Alipour and Maria Namjoo Baqhini reviewed and edited the original draft. All authors contributed to writing the draft and critical review.

## Funding

No funding was received for this research.

## Data Availability

The data used to support the findings of this study are available on request from the corresponding author.
